# Supplementary material for: Sequence and structural determinants of RNAPII CTD phase-separation and phosphorylation by CDK7
Source: Nat Commun. 2024 Oct 24;15:9163. doi: 10.1038/s41467-024-53305-2 (PMC11502803; doi:10.1038/s41467-024-53305-2)
Supplement: Supplementary file 1 — Supplementary Information [file 41467_2024_53305_MOESM1_ESM.pdf]

Supplementary Information

for

Title: Sequence and Structural Determinants of RNAPII CTD Phase-Separation and Phosphorylation by CDK7

Authors: Katerina Linhartova[1,2], Francesco Luca Falginella[1†], Martin Matl[1†], Marek Sebesta[1]*, Robert Vacha[1]* and Richard Stefl[1,2]*

Affiliations:

[1]CEITEC - Central European Institute of Technology, Masaryk University; Brno, Czechia.

[2]National Centre for Biomolecular Research, Faculty of Science, Masaryk University; Brno, Czechia.

† These authors contributed equally to this work

*Correspondence to: Email: richard.stefl@ceitec.muni.cz; robert.vacha@ceitec.muni.cz; marek.sebesta@ceitec.muni.cz

Supplementary Information includes:

Supplementary Figures 1 to 14
Supplementary Tables 1 to 4
Supplementary Methods
Supplementary References

Supplementary Figures

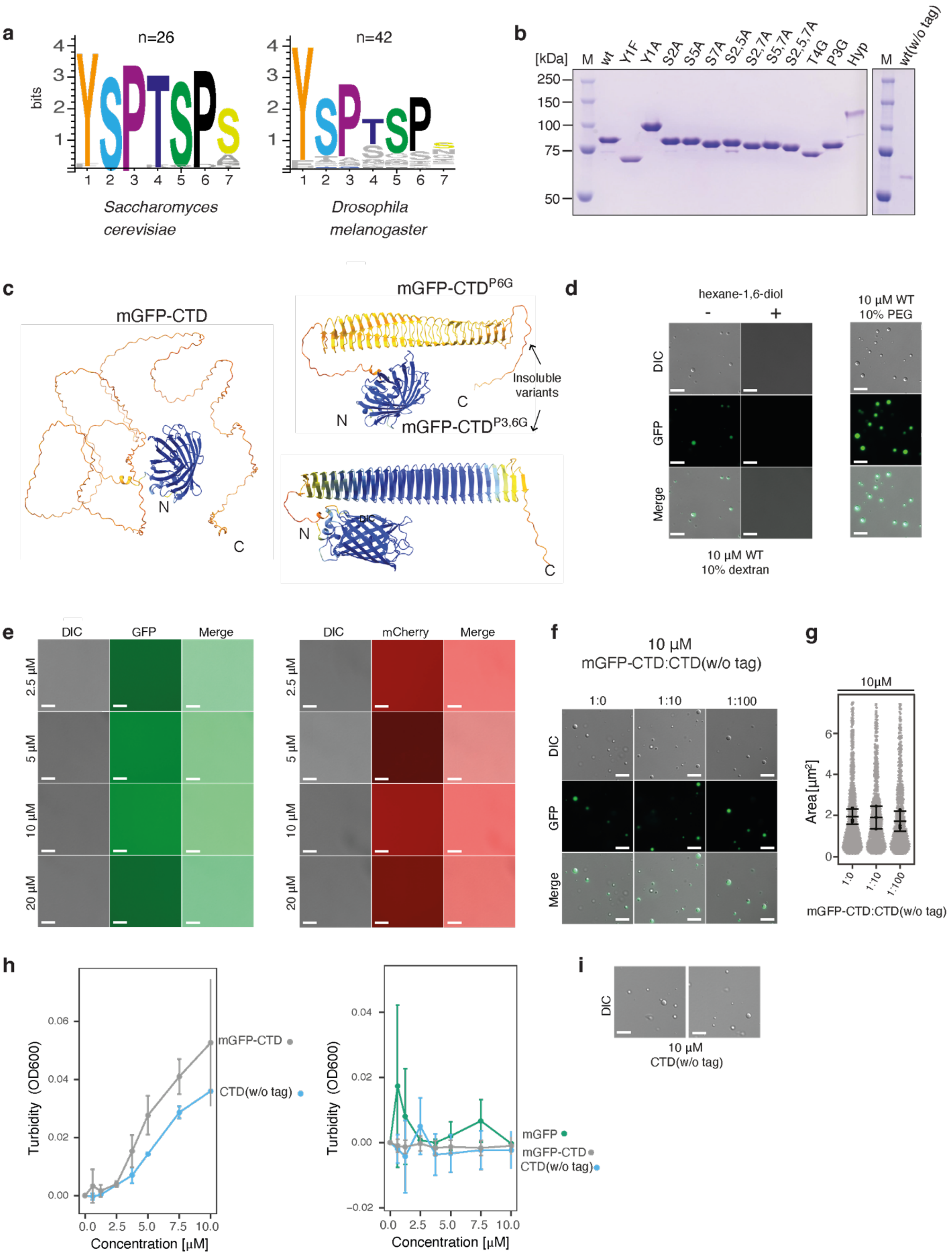

Supplementary Figure 1. (a) Sequence logo of the CTD heptad from *Saccharomyces cerevisiae (left)* and *Drosophila melanogaster (right)*, *n* = number of heptad repeats. **(b)** A scan of SDS-PAGE gel showing all RNAPII CTD variants tagged with mGFP, as well as the untagged CTD used in this study. **(c)** AlphaFold2 structure prediction of mGFP-CTD, mGFP-CTD^{P6G}, and mGFP-CTD^{P3,6G}. The generated models are colored according to the calculated per-residue estimate of confidence – pLDDT, model confidence: dark blue, very high (pLDDT > 90); light blue, confident (90 > pLDDT > 70); yellow, low (70 > pLDDT > 50); red, very low (pLDDT < 50). Images of the structures were generated in ChimeraX.[1] (**d,** *left*) Representative micrographs from at least three LLPS assays with 10 μM mGFP-CTD in 10% dextran 30 minutes after the LLPS initiation in the absence (-) and presence (+) of hexane-1,6-diol, respectively. (**d,** *right*) Representative micrographs from at least two LLPS assays with 10 μM mGFP-CTD in 10% PEG. **(e)** Representative micrographs of LLPS assay with the mGFP and mCherry tags alone. (*) Intensity of micrographs was uniformly enhanced in Fiji[2] for better visibility. **(f)** Representative micrographs from three independent LLPS assays with 10 μM mGFP-CTD and 10 μM CTD (w/o tag) at ratios 1:0, 1:10, and 1:100, respectively. **(g)** Quantifications of the experiments shown in **(f)**. Each dot represents a detected droplet, the black dots indicate median of droplet size per measurement (n=3). The black line and the error bar represent the mean value ± SD of the three medians. **(h)** Comparison of the turbidity assay measured at 600 nm for mGFP-CTD and CTD (w/o tag) at different concentrations in the presence of 10% dextran (*left*), the mGFP tag alone in the presence of 10% dextran, and mGFP-CTD and CTD (w/o tag) in the absence of 10% dextran (*right*). Data points and error bars represent mean ± SD from three independent LLPS assays. **(i)** Representative micrographs of two independent LLPS assays with 10 μM CTD. Panels **d**, **e**, **f**, **i**: DIC = Differential interference contrast, GFP/mCherry = Green/Red fluorescence channels, Merge = overlay of DIC and GFP/mCherry fluorescence channels. Scale bar = 10 μm. All experiments presented in panels **f-h** were done in triplicates. Source data are provided as a Source Data file.

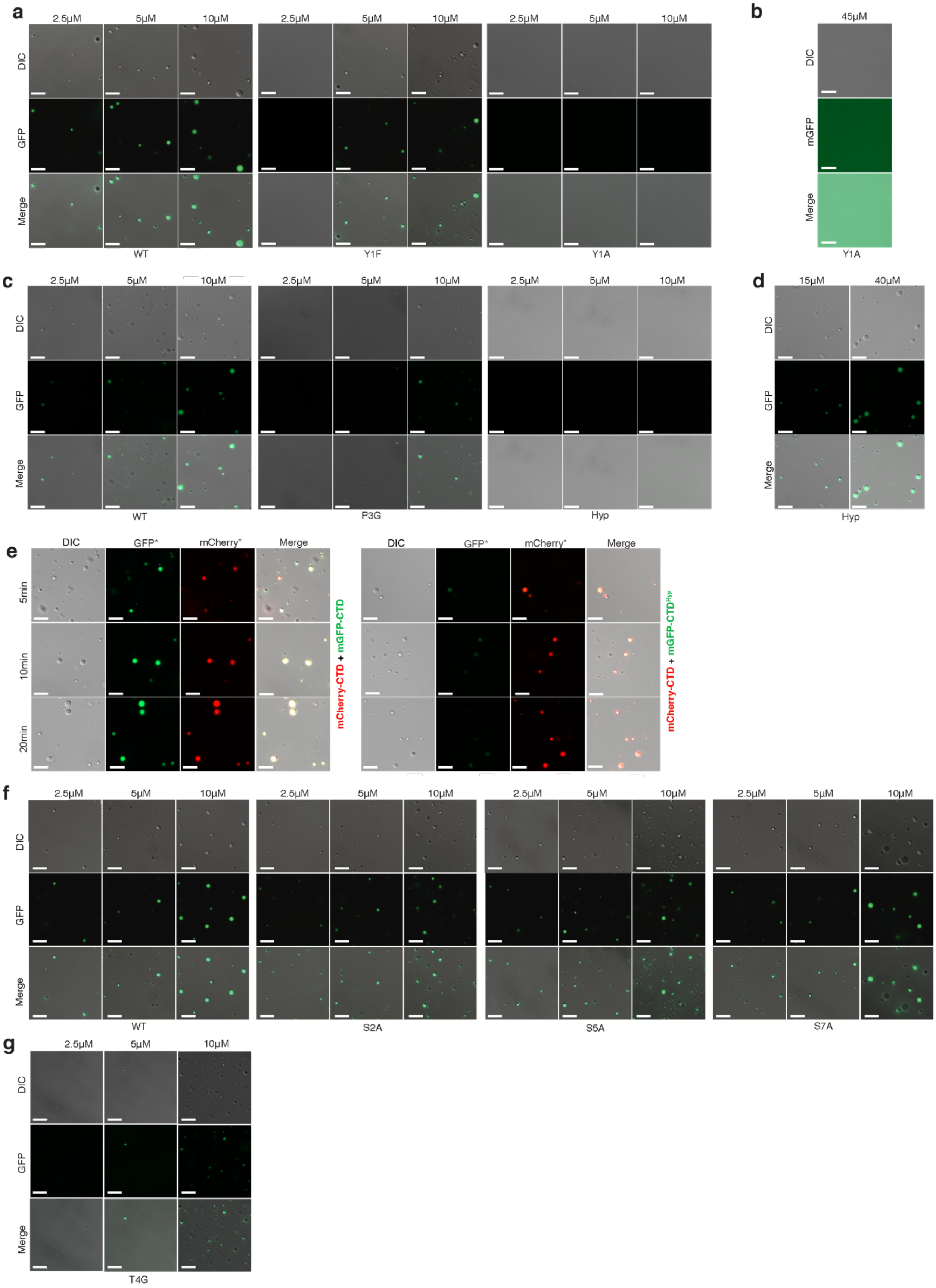
a
b
c
d
e
f
g
2.5µM
5µM
10µM
45µM
15µM
40µM
DIC
GFP
mGFP
Merge
mCherry
WT
Y1F
Y1A
P3G
Hyp
S2A
S5A
S7A
T4G
5min
10min
20min
mCherry-CTD + mGFP-CTD
mCherry-CTD + mGFP-CTD^Hyp

Supplementary Figure 2. The importance of human RNAPII CTD residues in phase-separation. Representative micrographs for the analyses depicted in **Figure 1**. **(a)** Representative micrographs from three independent in vitro LLPS assays with mGFP-CTD, mGFP-CTD^{Y1F}, and mGFP-CTD^{Y1A}, respectively, analysis depicted in **Figure 1d**. **(b)** Representative micrographs from two independent in vitro LLPS assays with mGFP-CTD^{Y1A} at a concentration of 45 μM. **(c)** Representative micrographs from three independent in vitro LLPS assays with mGFP-CTD, mGFP-CTD^{P3G}, and mGFP-CTD^{Hyp}, respectively, analysis depicted in **Figure 1e. (d)** Representative micrographs from in vitro LLPS assay with mGFP-CTD^{Hyp} (at 15 μM and 40 μM). **(e)** Representative micrographs from three independent in vitro LLPS mixing assays with mGFP-CTD and mCherry-CTD, and mGFP-CTD^{Hyp} and mCherry-CTD, respectively, analysis depicted in **Figure 1f** and **g**. (*) The intensity of all fluorescence micrographs was uniformly enhanced in Fiji[2] for better visibility. **(f)** Representative micrographs from three independent in vitro LLPS assays with mGFP-CTD^{S2A}, mGFP-CTD^{S5A}, and mGFP-CTD^{S7A}, respectively, analysis depicted in **Figure 1h. (g)** Representative micrographs from three independent in vitro LLPS assays with mGFP-CTD and mGFP-CTD^{T4G}, analysis depicted in **Figure 1i**. Panels **a-g**: DIC = Differential interference contrast, GFP/mCherry = Green/Red fluorescence channel, Merge = overlay of DIC and GFP/mCherry channel. Scale bar = 10 μm.

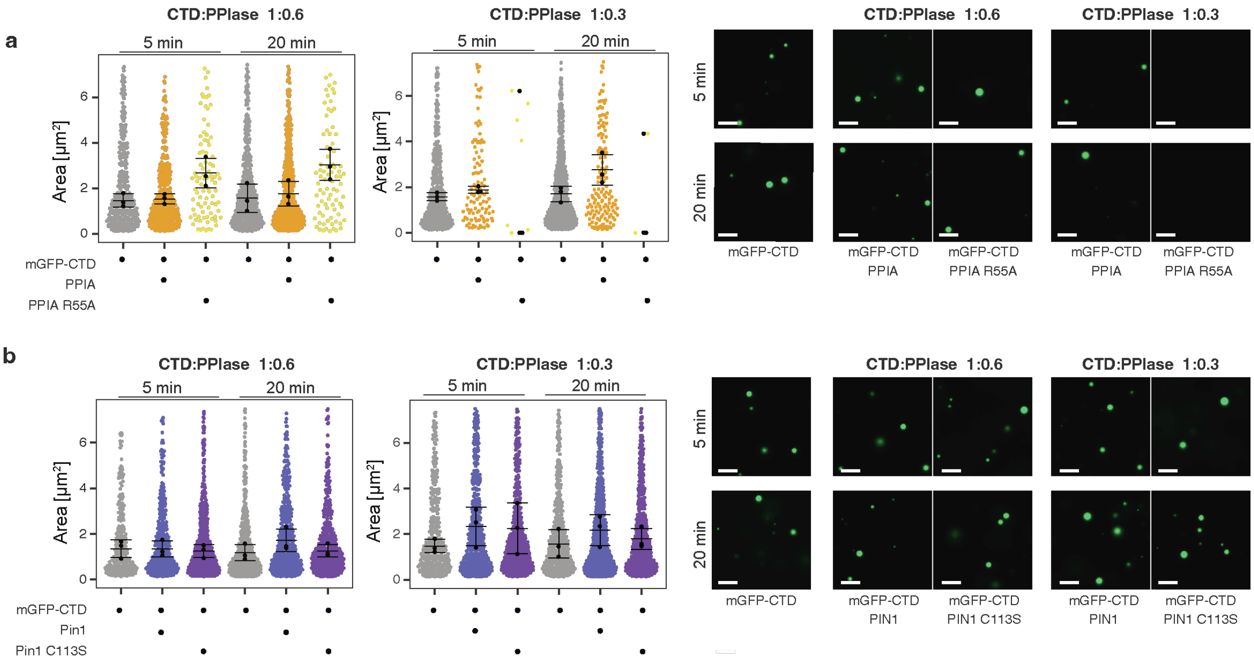

Supplementary Figure 3. (**a** left) Quantification of the in vitro LLPS assay with mGFP-CTD and mGFP-CTD in the presence of PPIA and the PPIA^{R55A} variant, respectively, at ratios of 1:0.6, 1:0.3 (mGFP-CTD to PPIA/ PPIA^{R55A}). Each dot represents a detected droplet, the black dots indicate the median of droplet size per measurement (n=3). The black line and error bars represent the mean ± SD of the three medians. (**a** right) Representative micrographs from three independent LLPS assays of mGFP-CTD, mGFP-CTD with PPIA, and mGFP-CTD with PPIA^{R55A} (right). Scale bar = 10 µm. (**b** *left*) Quantification of the in vitro LLPS assay with mGFP-CTD in the presence of PIN1 and the PIN1^{C113S} variant, respectively, at ratios of 1:0.6, 1:0.3 (5µM mGFP-CTD, and 160 µM and 80µM PPIase, respectively) (*left*). Each dot represents a detected droplet, the black dots indicate the median of droplet size per measurement (n=3). The black line and error bars represent the mean ± SD of the three medians. (**b** *right*) Representative micrograph from three independent LLPS assays with mGFP-CTD, mGFP-CTD with PIN1 and mGFP-CTD with PIN1^{C113S}, respectively, at molar ratios of 1:0.6, 1:0.3 (mGFP-CTD to PIN1/ PIN1^{C113S}). Scale bar = 10 µm. Intensity of all micrographs was uniformly enhanced in Fiji [2] for better visibility. Source data are provided as a Source Data file.

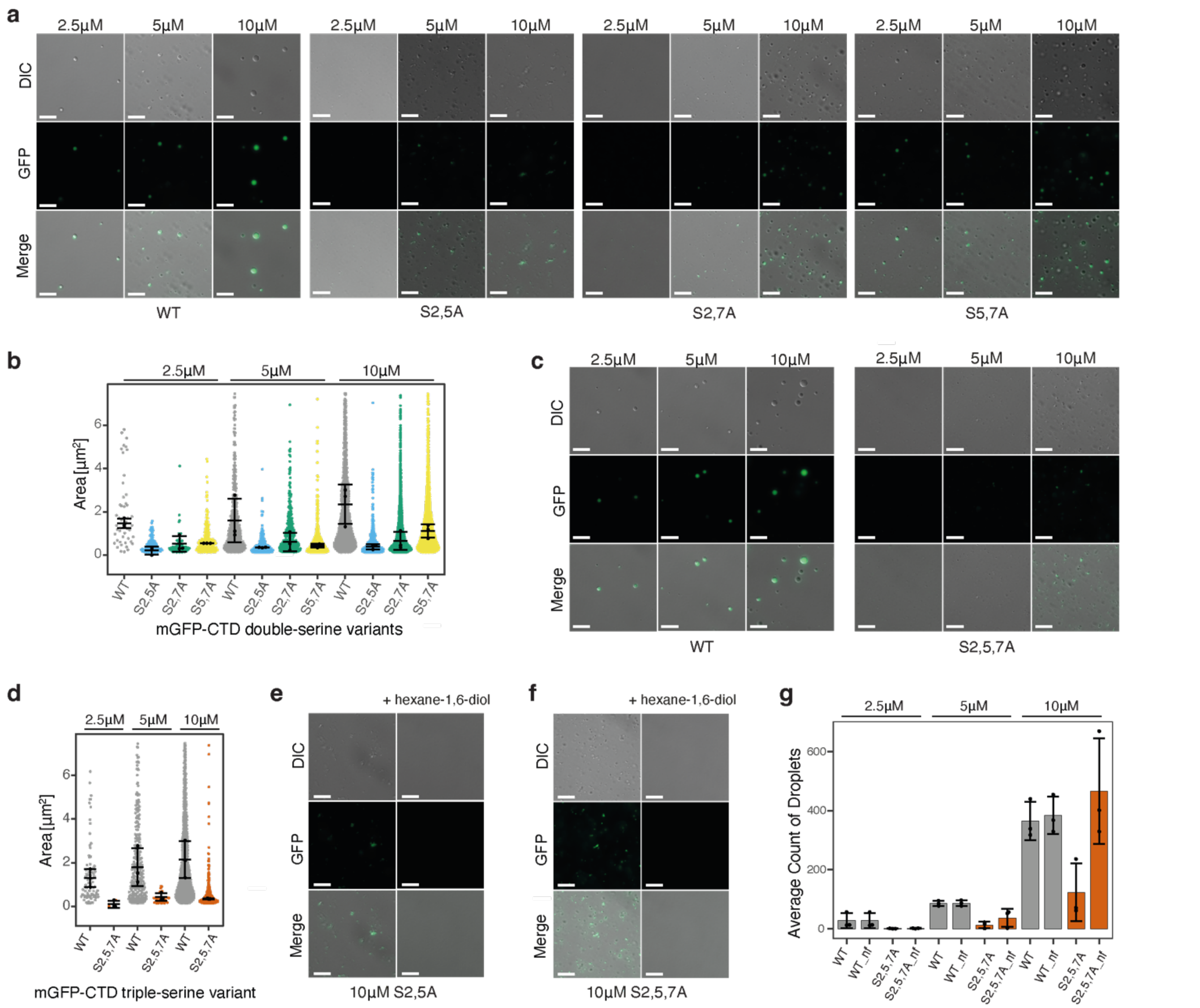

Supplementary Figure 4. The importance of RNAPII CTD residues in phase-separation. **(a)** Representative micrographs from three independent in vitro LLPS assays with mGFP-CTD, mGFP-CTD^{S2,5A}, mGFP-CTD^{S2,7A}, and mGFP-CTD^{S5,7A}. **(b)** Quantification of LLPS assay depicted in **(a)**. Each dot represents a detected droplet, the black dots indicate the median of droplet size per measurement (n=3). The black line and error bars represent the mean ± SD of the three medians. **(c)** Representative micrographs from three independent in vitro LLPS assay of mGFP-CTD, and mGFP-CTD^{S2,5,7A}. Scale bar = 10 µm. **(d)** Quantification of the LLPS assay depicted in **(c)**. Each dot represents a detected droplet, the black dots indicate the median of droplet size per measurement (n=3). The black line and error bars represent the mean ± SD of the three medians. Representative micrographs from three independent LLPS assays with 10 µM mGFP-CTD^{S2,5A} **(e)** and mGFP-CTD^{S2,5,7A} **(f)** in the presence and absence of hexane-1,6-diol, respectively. Scale bar = 10 µm. **(g)** Comparison of the analyses of the average droplet count for the in vitro LLPS assay with mGFP-CTD and mGFP-CTD^{S2,5,7A} (depicted in panel (c)) with and without (nf) using the eccentricity filter, respectively. The black dots indicate the sum of the droplets per measurement (n=3). The bars and the error bars represent the mean ± SD of the three measurements. Panels **a**, **c**, **e**, **f**: DIC = Differential interference contrast, GFP = Green Fluorescence channel, Merge = overlay of DIC and GFP channel. Scale bar = 10 µm. Source data are provided as a Source Data file.

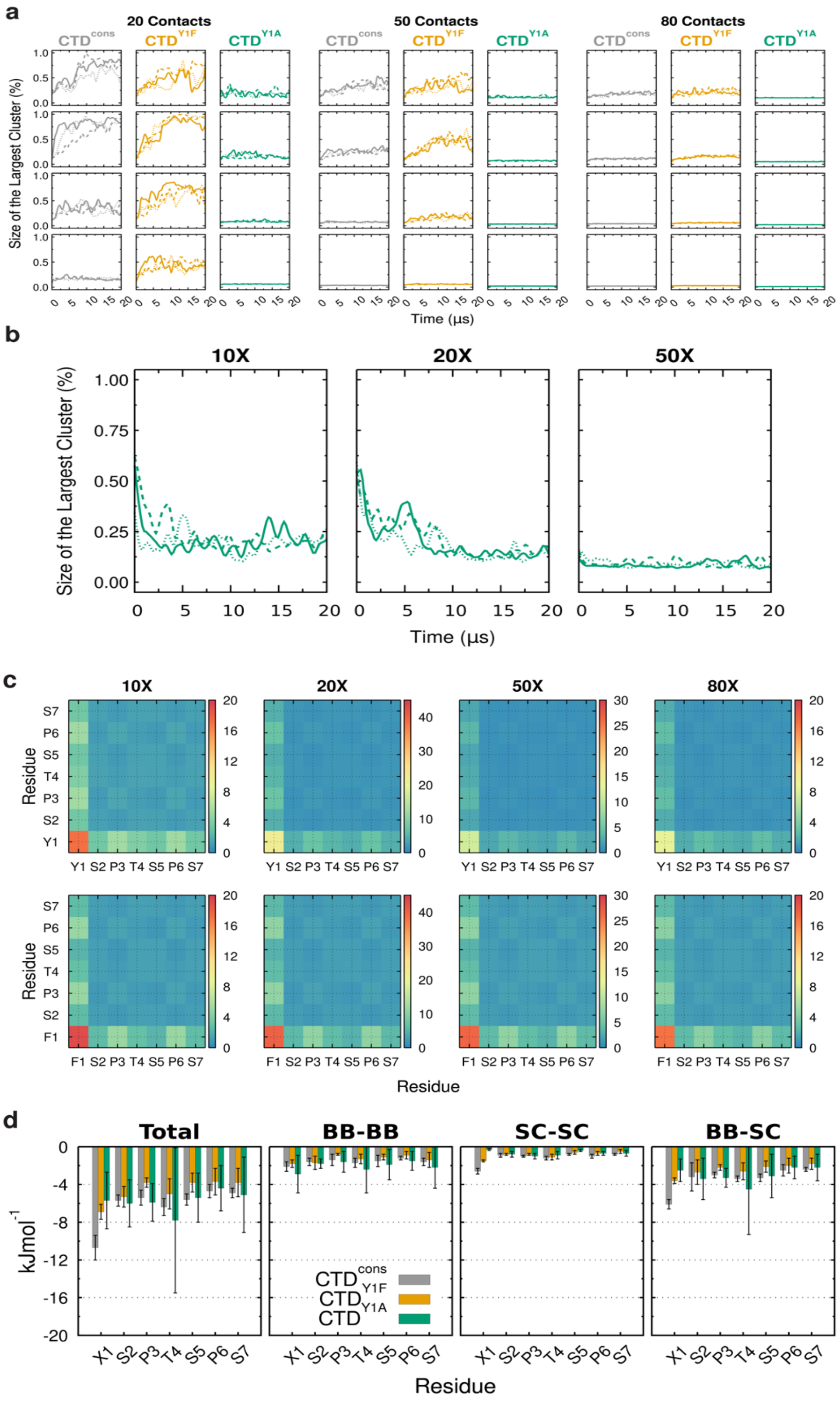

a
20 Contacts
50 Contacts
80 Contacts
CTD cons
CTD Y1F
CTD Y1A
Size of the Largest Cluster (%)
Time (μs)
b
10X
20X
50X
Size of the Largest Cluster (%)
Time (μs)
c
10X
20X
50X
80X
Residue
Y1 S2 P3 T4 S5 P6 S7
F1 S2 P3 T4 S5 P6 S7
d
Total
BB-BB
SC-SC
BB-SC
kJmol-1
CTD cons
CTD Y1F
CTD Y1A
X1 S2 P3 T4 S5 P6 S7
Residue

Supplementary Figure 5. **(a)** Time series of the size of the largest protein cluster over the entire 20 μs of CG condensed phase simulations for CTD^{cons} (gray), as well as the CTD^{Y1F} (orange) and CTD^{Y1A} (green) variants. The data are shown for three contact cutoffs (see Methods). The graphs, arranged from top to bottom, display cluster sizes for 10, 20, 50, and 80 CTD molecules. The three replicas are displayed using solid, dashed, and dotted lines, respectively. **(b)** Same as in **(a)** except for the initial configurations, which consisted of preformed condensates of CTD^{cons}. Only results for the least conservative contact cutoff are shown (*i.e.*, 20 contacts). From left to right, the cluster sizes for 10, 20, and 50 CTD^{Y1A} molecules are displayed. **(c)** Intermolecular contacts grouped by residue type for CTD^{cons} and the CTD^{Y1F} variant in condensed phase CG simulations. The data represent an average over three replicas and all CTD molecules. The SDs for 10, 20, 50, and 80 CTD molecules range from 0.135 to 4.403, 0.067 to 1.859, 0.023 to 1.217, and 0.009 to 0.548, respectively. **(d)** Intermolecular interaction energy contributions grouped by residue type in all-atom simulations. BB, SC, and X respectively stand for backbone, side chain, and one among Y, F, and A. The shown values represent the mean ± SD over all three replicas and CTD copies. Source data are provided as a Source Data file.

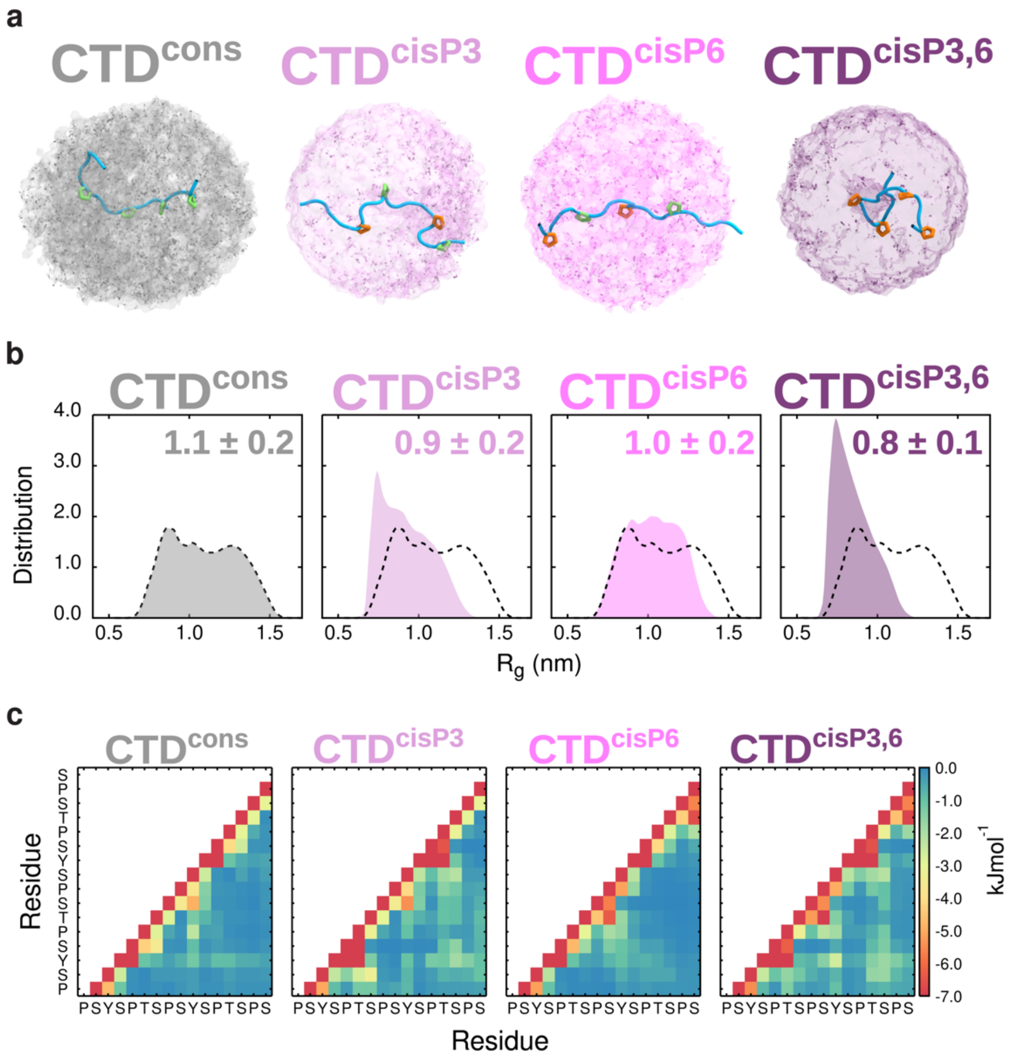

Supplementary Figure 6. MD simulations of RNAPII CTD constructs. **(a)**, **(b)**, and **(c)** respectively expand the data shown in **Figure 2c**, **d**, and **e**, to include single *cis* isomerization variant at P3 and P6. In **(c)**, SD ranges from 0.003 to 5.920. Source data are provided as a Source Data file.

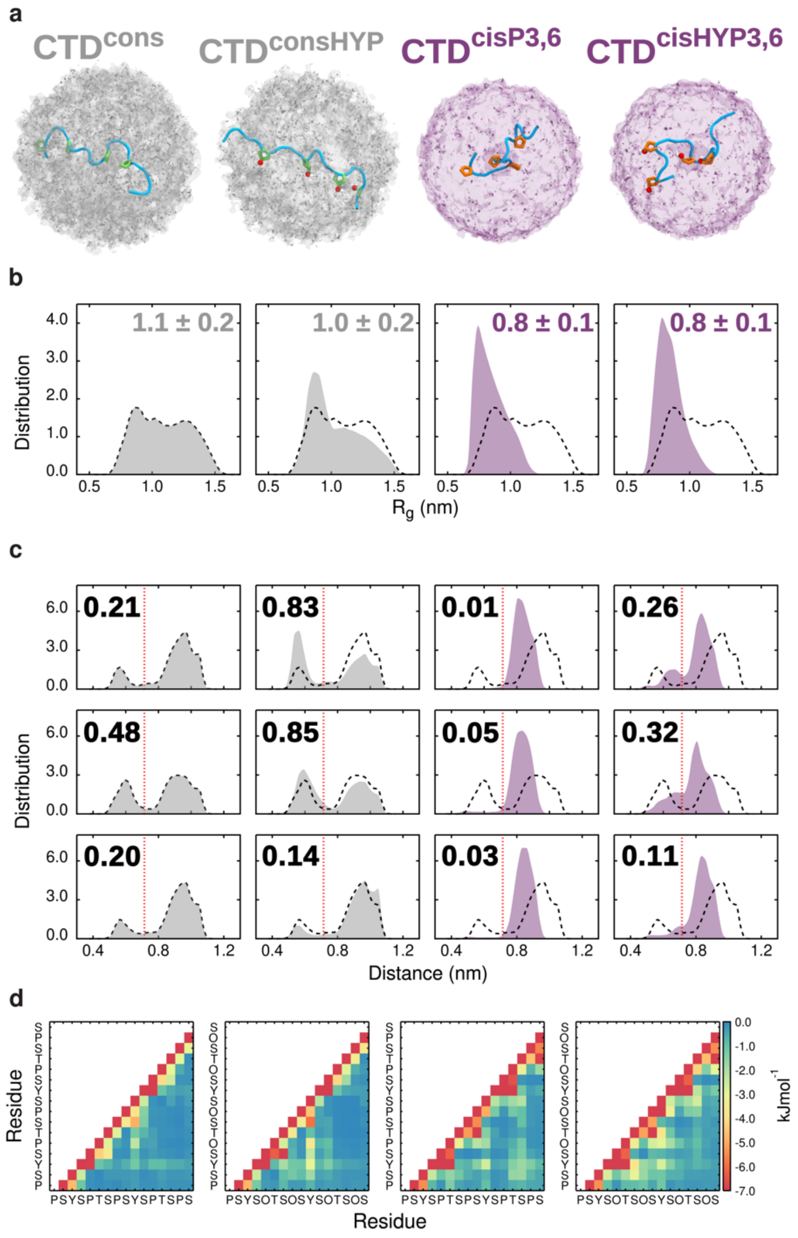

Supplementary Figure 7. (a), **(b)**, **(c)**, and **(d)** show a comparison between *cis* and *trans* isomerization variants of CTD constructs with proline or hydroxyproline, extending **Figure 2c**, **d**, **e**, and **g**, respectively. All panels follow the same columns order. In **(d)** hydroxyproline is coded by the letter O and the SD ranges from 0.003 to 7.297. Source data are provided as a Source Data file.

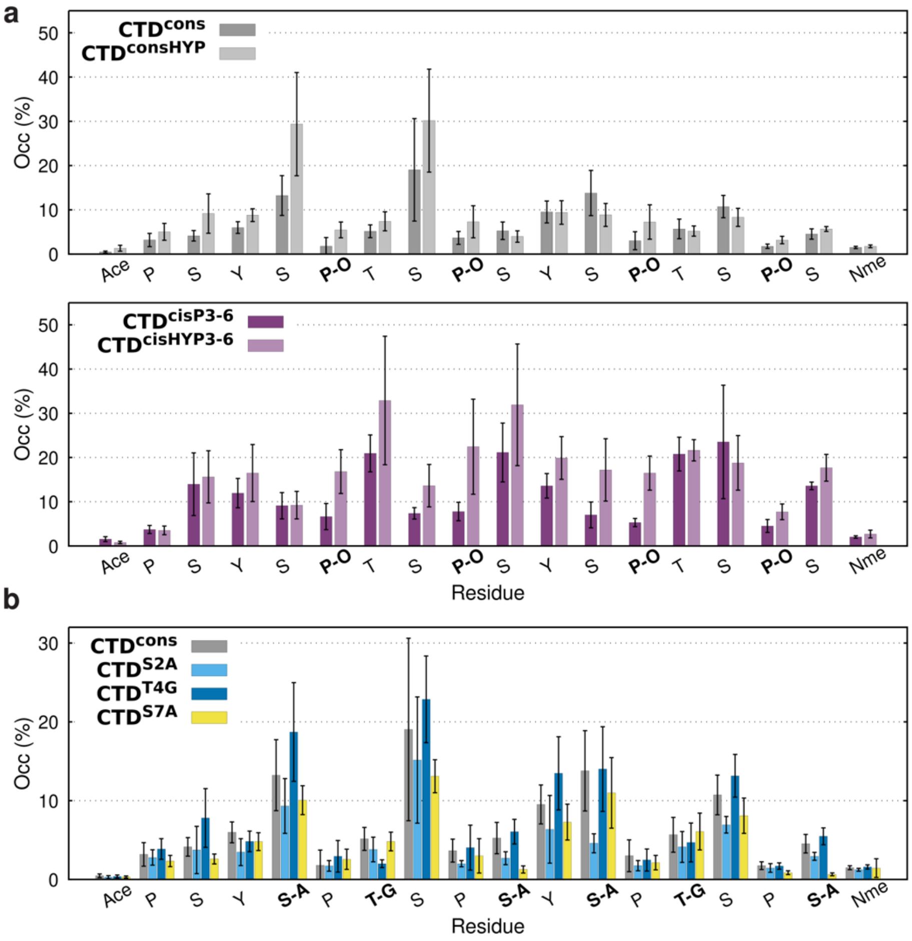

Supplementary Figure 8. Per-residue occupancy of intramolecular hydrogen bonds. **(a)** Occupancy (Occ) for *cis*/*trans* hydroxyproline variants (lighter hues) with respect to their proline counterparts (darker hues). Hydroxyproline is coded by the letter O. **(b)** Occupancy for CTD^{S2A}, CTD^{T4G}, and CTD^{S7A} with respect to CTD^{cons}. The results are shown for the full-length di-heptads (*i.e.*, including caps) and represent the mean ± SD over all three replicas and CTD copies. Intra-residue hydrogen bonds were excluded from the analysis. The mutated sites are shown in bold. Source data are provided as a Source Data file.

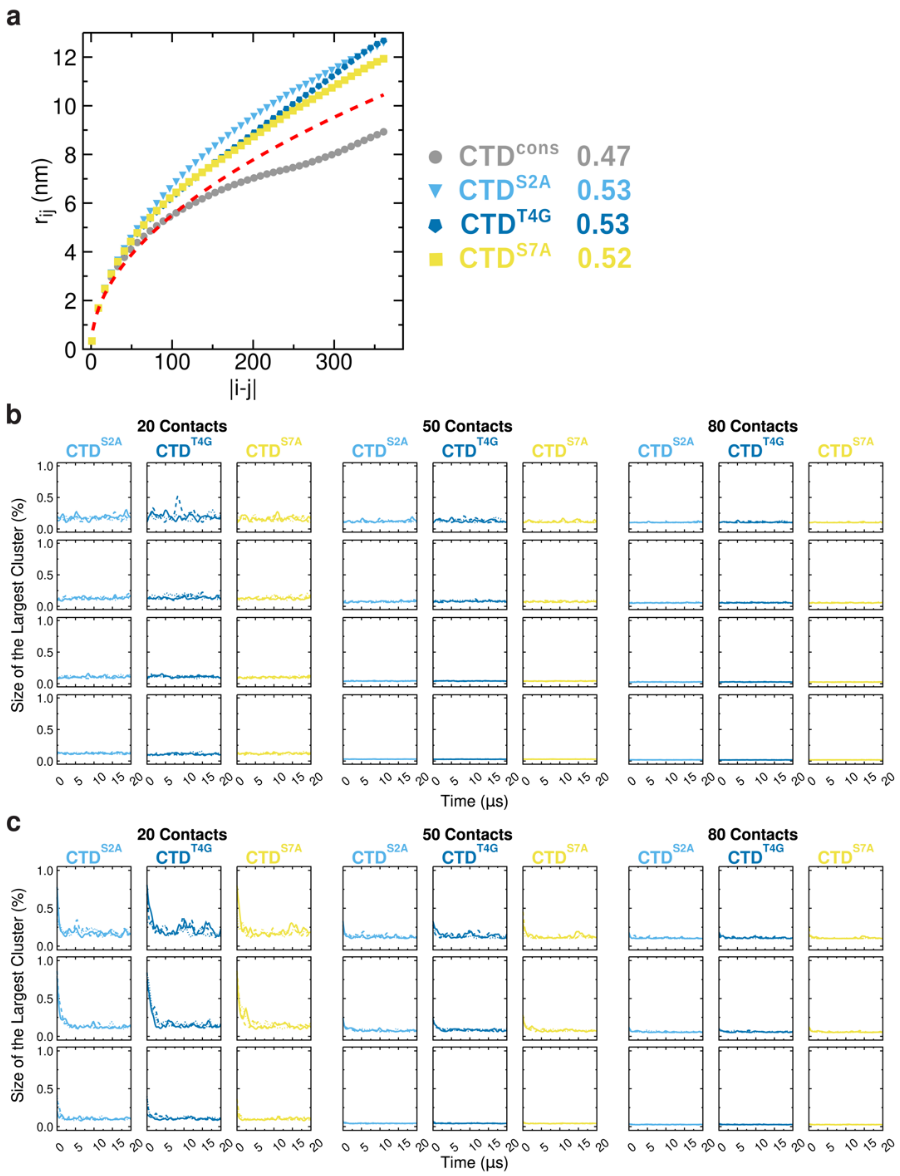

Supplementary Figure 9. (a) Average intramolecular distance (r_{ij}) as a function of residue separation (|*i*-*j*|) for CTD^{cons} (gray circles) and variants CTD^{S2A} (light blue inverted triangles), CTD^{T4G} (dark blue pentagons), and CTD^{S7A} (yellow squares) in CG single chain simulations. The values of the Flory scaling exponent (ν; see Methods for details) averaged over chain separation > 200 are shown on the right (maximum standard deviation of 0.003). The scaling behavior of an ideal chain ($\nu = 0.5$) is depicted as the red dashed line showing a threshold between extended conformations ($\nu > 0.5$) and compact conformations ($\nu < 0.5$) with potential for phase-separation. **(b)** Time series of the size of the largest protein cluster over the entire 20 µs of CG condensed phase simulations for variants CTD^{S2A} (light blue), CTD^{T4G} (dark blue), and CTD^{S7A} (yellow). The data are shown for three contact cutoffs (see Methods). The graphs, arranged from top to bottom, display cluster sizes for 10, 20, 50, and 80 CTD molecules. The three replicas are displayed using solid, dashed, and dotted lines, respectively. **(c)** Same as in **(b)** except for the initial configurations, which consisted of preformed condensates of CTD^{cons}. The graphs, arranged from top to bottom, display cluster sizes for 10, 20, and 50 CTD molecules. Source data are provided as a Source Data file.

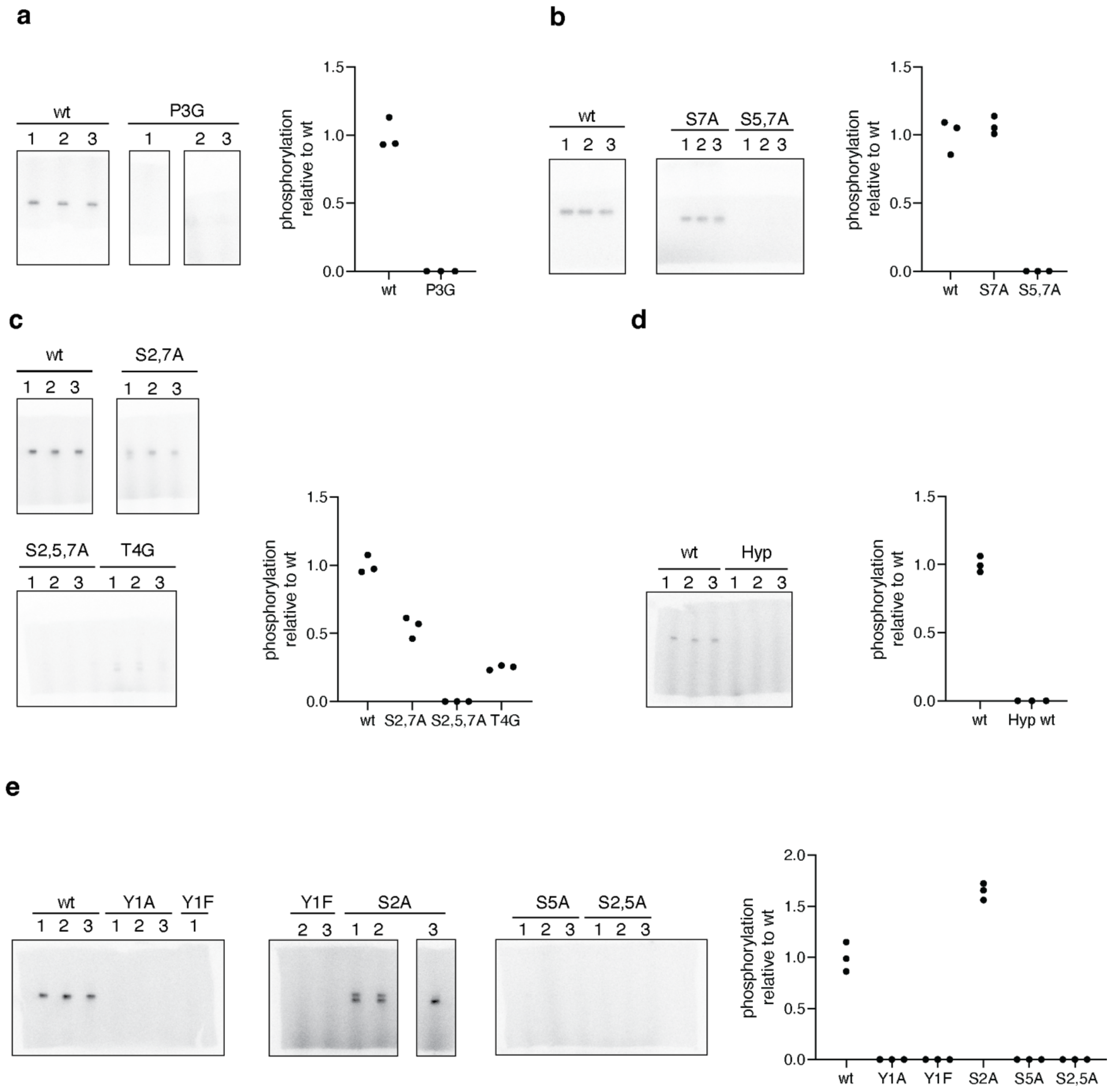

Supplementary Figure 10. The impact of CTD heptad residues on phosphorylation by the CDK7 complex and the analysis of CTD phosphorylation under phase-separation conditions. **(a-e)** Scans of phosphor imager screens and the quantifications depicted in **Figure 3a**. The experiments used to construct **Figure 3a** were performed in the same manner, but at different time points. Left: scanned phosphor imager screens. Right: plotted signal intensities for variant proteins relative to the mean of wild-type CTD, to account for radioactive ATP decay. Source data are provided as a Source Data file.

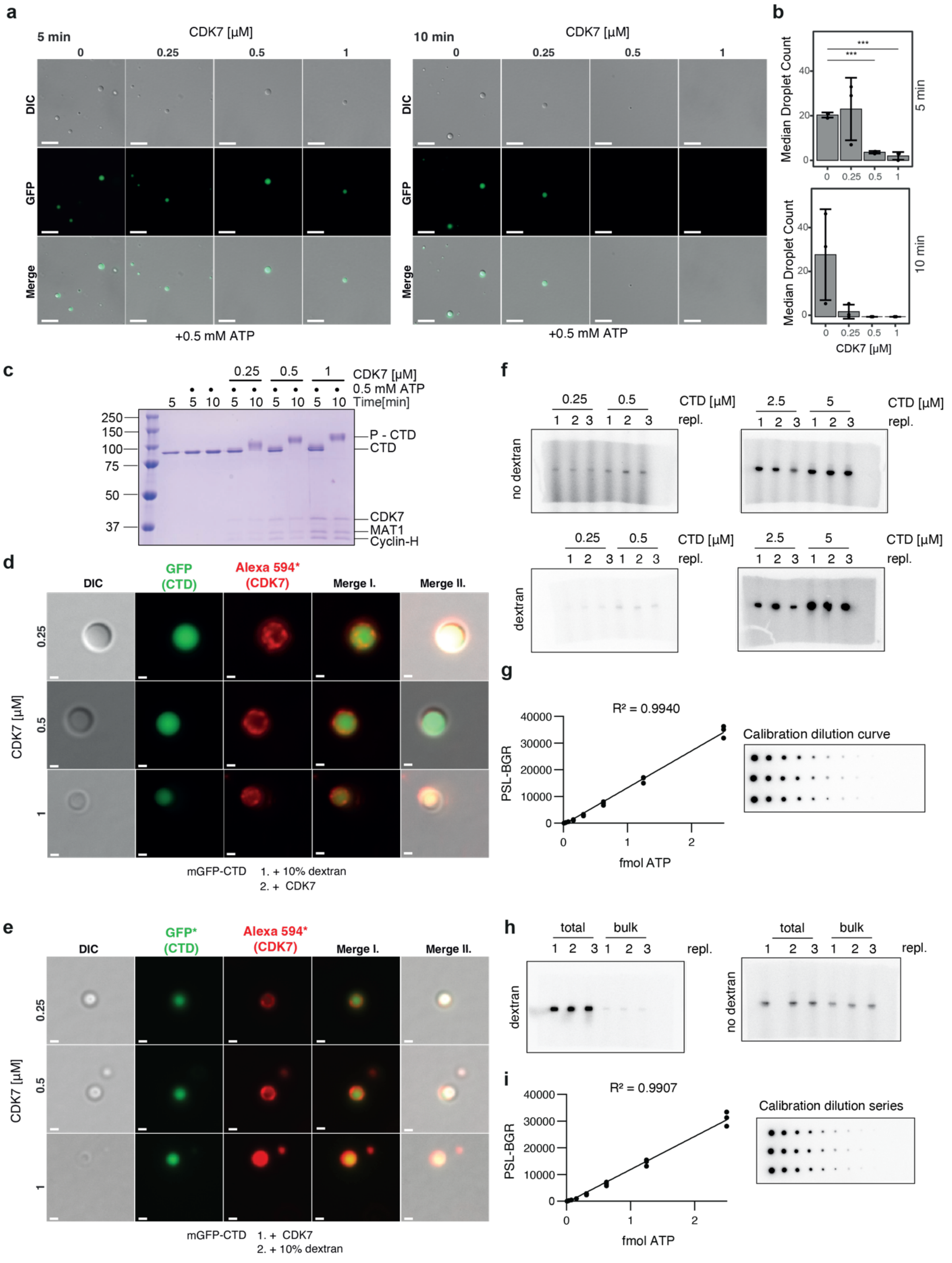
a
5 min
CDK7 [µM]
0
0.25
0.5
1
10 min
DIC
GFP
Merge
+0.5 mM ATP
b
Median Droplet Count
5 min
10 min
CDK7 [µM]
c
CDK7 [µM]
0.5 mM ATP
Time[min]
250
150
100
75
50
37
P - CTD
CTD
CDK7
MAT1
Cyclin-H
d
GFP (CTD)
Alexa 594* (CDK7)
Merge I.
Merge II.
mGFP-CTD 1. + 10% dextran
2. + CDK7
e
GFP* (CTD)
mGFP-CTD 1. + CDK7
2. + 10% dextran
f
CTD [µM]
repl.
no dextran
dextran
g
R² = 0.9940
Calibration dilution curve
PSL-BGR
fmol ATP
h
total
bulk
i
R² = 0.9907
Calibration dilution series

Supplementary Figure 11. The impact of CTD heptad residues on phosphorylation by the CDK7 complex and the analysis of CTD phosphorylation under phase-separation conditions. **(a)** Representative micrographs from three independent in vitro LLPS kinase assays depicted in **Figure 3b** and **c**. Scale bar = 10 µm. DIC = Differential interference contrast, GFP = Green Fluorescence channel, Merge = Overlay of DIC and GFP channels. **(b)** Droplet count quantifications from the data depicted in **Figure 3b** and **c**. The black dots indicate the median of droplet count per measurement from five images (n=3). The black lines and error bars represent the mean ± SD of the three medians. Statistical comparisons of means were determined via an unpaired, two-sided t-test (see Methods: FM-image analysis), only $p \leq 0.05$ are depicted. p-values: **(top)** ***=0.000223, ***= 0.00025823, respectively. **(c)** A scan of SDS-PAGE gel from kinase reaction samples used for the fluorescence microscopy measurements depicted in **Figure 3b** and **c**. Representative micrographs from three independent in vitro LLPS co-localization assays of mGFP-CTD (GFP) and different concentration of the CDK7 complex (Alexa 594) depicted in **Figure 3f (d)** and with incubation of the mGFP-CTD (GFP) and the CDK7 complex (Alexa 594) prior the start of the phase-separation **(e)**. Scale bar = 1 µm. (*) The intensity of all fluorescence micrographs was uniformly enhanced in Fiji[2] for better visibility. Merge I. = overlay of GFP and Alexa 594 channels, Merge II. = overlay of GFP and Alexa 594 channels with DIC channel. **(f)** Scanned phosphorimager screens of γ-[^{32}P]-ATP kinase reactions analyzed on SDS-PAGE gels, as quantified in **Figure 3h** (n=3). **(g)** Scanned phosphor imager screen showing the dilution calibration curve used to relate signal intensity to ATP amount for experiment in **Figure 3h**. The two-fold, ten-step dilution series was performed in triplicates and R^2 values are shown above the graph. PSL – photostimulated luminescence, BGR – background. **(h)** Scanned phosphor imager screens of γ-[^{32}P]-ATP kinase reactions analyzed on SDS-PAGE gels, as quantified in **Figure 3i** (n=3). Note that the reactions containing 10% dextran were diluted 10-fold to avoid oversaturation of the phosphor imager (see Methods). **(i)** Scanned phosphor imager screen showing the dilution calibration curve used to relate signal intensity to ATP amount for experiment in **Figure 3i**. The two-fold, ten-step dilution series was performed in triplicates and R2 values are shown above the graph. PSL – photostimulated luminescence, BGR – background. Source data are provided as a Source Data file.

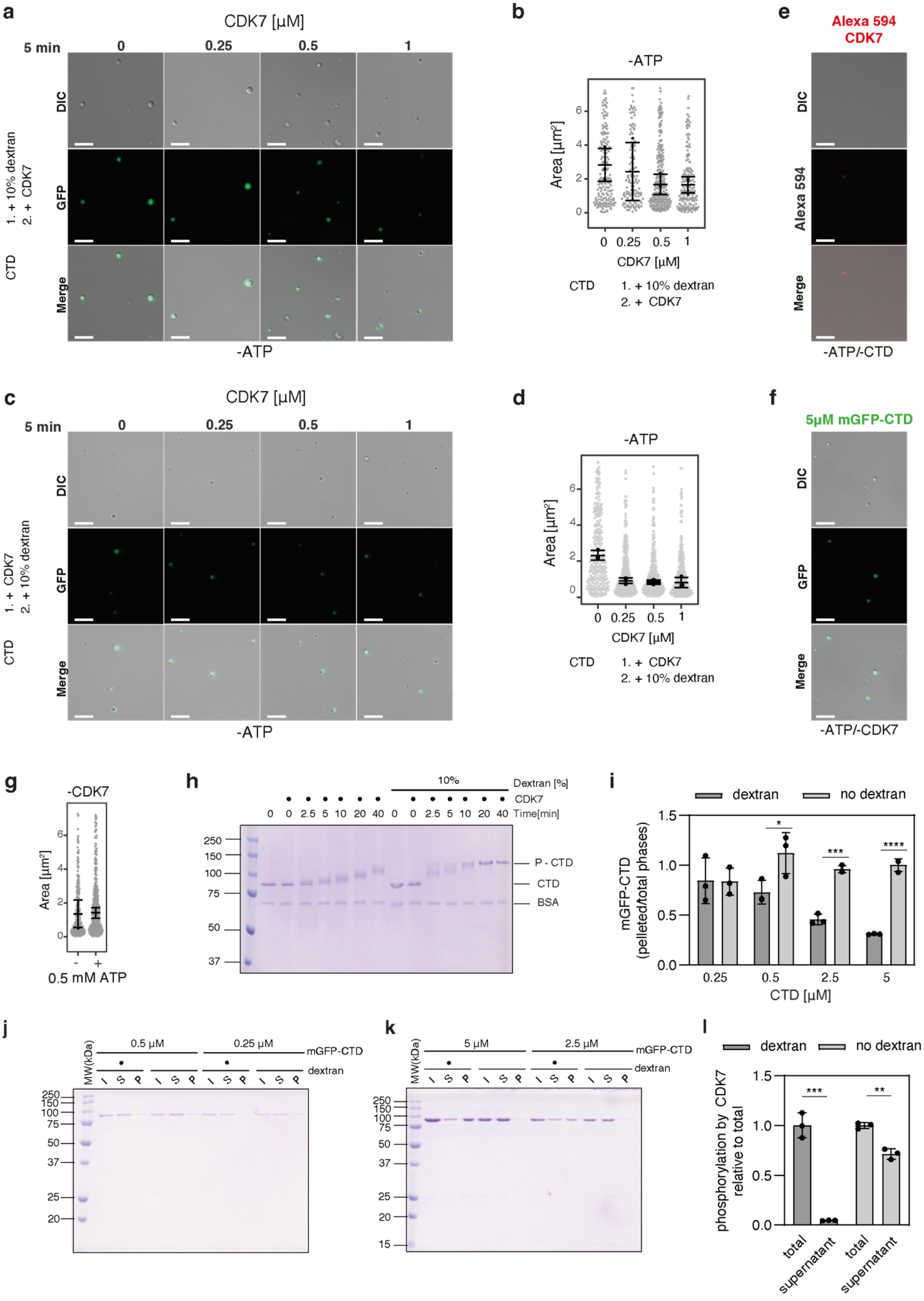
a
CDK7 [μM]
5 min
0
0.25
0.5
1
DIC
GFP
Merge
1. + 10% dextran
2. + CDK7
CTD
-ATP
b
-ATP
Area [μm²]
CDK7 [μM]
CTD
1. + 10% dextran
2. + CDK7
e
Alexa 594
CDK7
DIC
Alexa 594
Merge
-ATP/-CTD
c
CDK7 [μM]
5 min
0
0.25
0.5
1
DIC
GFP
Merge
1. + CDK7
2. + 10% dextran
CTD
-ATP
d
-ATP
Area [μm²]
CDK7 [μM]
CTD
1. + CDK7
2. + 10% dextran
f
5μM mGFP-CTD
DIC
GFP
Merge
-ATP/-CDK7
g
-CDK7
Area [μm²]
0.5 mM ATP
h
10%
Dextran [%]
CDK7
Time[min]
P - CTD
CTD
BSA
i
dextran
no dextran
mGFP-CTD (pelleted/total phases)
CTD [μM]
j
0.5 μM
0.25 μM
mGFP-CTD
dextran
MW(kDa)
k
5 μM
2.5 μM
mGFP-CTD
dextran
MW(kDa)
l
dextran
no dextran
phosphorylation by CDK7 relative to total
total
supernatant

Supplementary Figure 12. The impact of CTD heptad residues on phosphorylation by the CDK7 complex and the analysis of CTD phosphorylation under phase-separation conditions. **(a)** Representative micrographs from three independent in vitro LLPS kinase assays with 5 µM mGFP-CTD (green) with different concentrations of the CDK7 complex. Scale bar = 10 µm. **(b)** Quantifications of the in vitro LLPS kinase assays in **(a)**. Each dot represents a detected droplet, black dots indicate the median of droplet size per measurement (n=3). The black lines and error bars represent the mean ± SD of the three medians.
(c) same as **(a)** but for mGFP-CTD incubated with the CDK7 complex prior to the start of phase-separation. **(d)** Quantifications of the in vitro LLPS kinase assays in **(c)**. Each dot represents a detected droplet, black dots indicate the median of droplet size per measurement (n=3). The black lines and error bars represent the mean ± SD of the three medians. **(e)** Representative micrographs of 1 µM CDK7 labeled with Alexa 594 (red) in 10% dextran. Scale bar =10 µm. **(f)** Representative micrographs of 5 µM mGFP-CTD (green) in 10% dextran and in the absence of the CDK7 complex and ATP. **(g)** Quantification of fluorescence images from the LLPS assays of 5 µM mGFP-CTD in the absence of ATP and the CDK7 complex depicted in **(f)** and with 0.5 mM ATP and in the absence of the CDK7 complex depicted in **Figure 3b**. Each dot represents a detected droplet, black dots indicate the median of droplet size per measurement (n=3). The black lines and error bars represent the mean ± SD of the three medians. **(h)** SDS-PAGE gel showing kinase reactions of 2.5 µM mGFP-CTD with 100 nM CDK7 complex and 0.5 mM ATP, incubated in the presence or absence of 10% dextran and quenched at different time points. (P-CTD) phosphorylated CTD, (BSA) Bovine serum albumin. **(i)** Quantification of the sedimentation assay of mGFP-CTD phase-separation at different concentrations in the presence and absence of 10% dextran (n=3). The mean ± SD of the ratio of pelleted versus total fractions is plotted. **(j)** and **(k)** Scans of representative SDS-PAGE gel for quantification of CTD phase-separation at different concentrations in **(i)**. **(i)** input, **(s)** supernatant, **(p)** pellet. **(l)** Sedimentation kinase assay (depicted in **Figure 3i**) displayed as normalized to the "total" levels of phosphorylation under the tested conditions. The total level of phosphorylation was set as baseline, from which the fraction of the bulk level of phosphorylation was derived. The mean ± SD are plotted (n=3). Panels **a**, **c**, **e**, **f**: DIC = differential interference contrast, GFP/Alexa594 = fluorescence channels, Merge = overlay of DIC and GFP/Alexa594 channel. Statistical significance for panels **i**,**l** was determined by unpaired, two-sided t-tests. P-values:**(i)** *= 0.045951, ***=0.000165, **** = 0.000050, **(l)** *** = 0.0002, ** = 0.0012

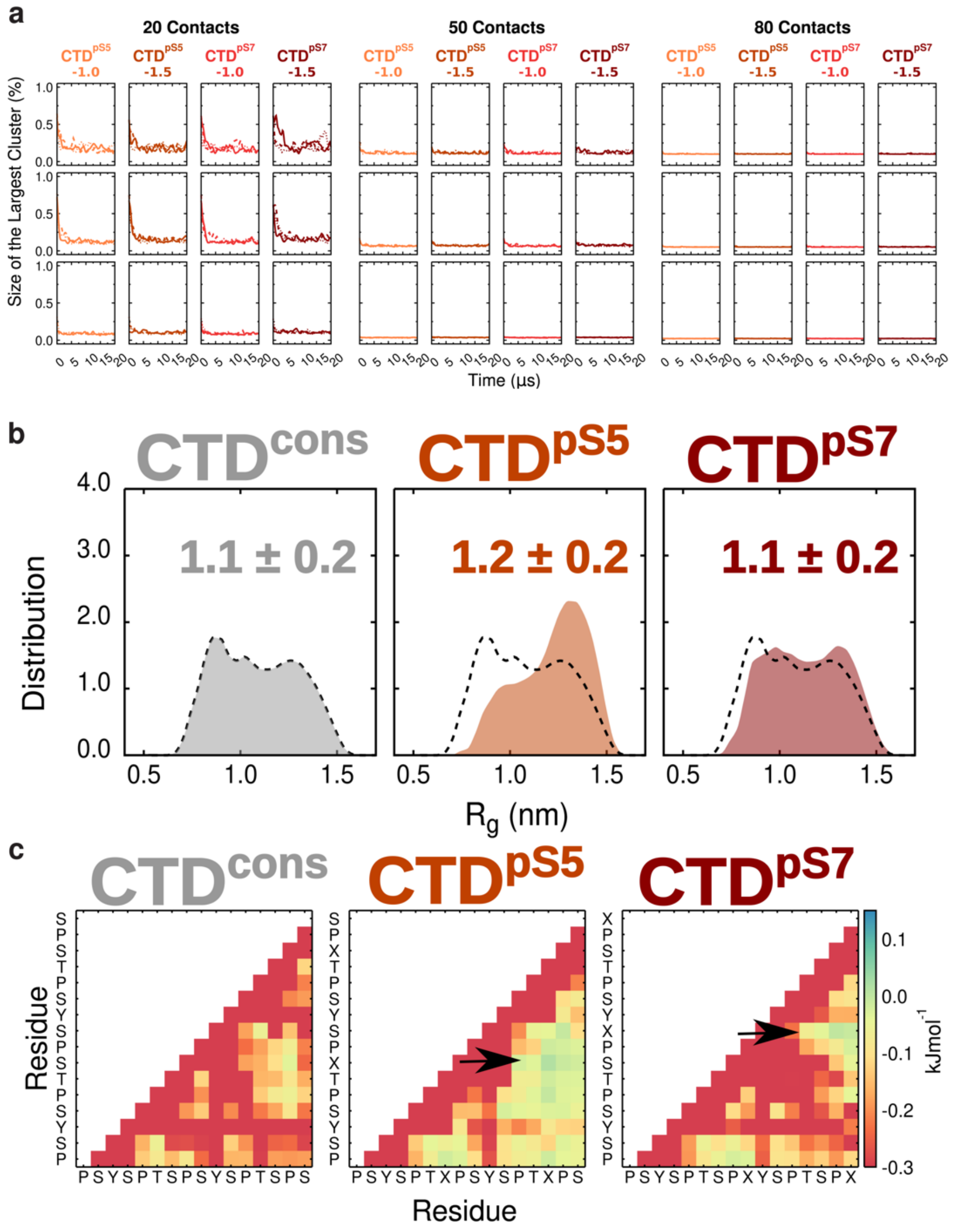

Supplementary Figure 13. (a) Time series of the size of the largest protein cluster over the entire 20 μs of condensed phase CG simulations for the CTD constructs phosphorylated at S5 (orange) and S7 (red), where the initial configurations consisted of preformed condensates of CTD^{cons}. The two charge states of the phosphate group are rendered with light (-1*e*) and dark (-1.5*e*) hues. The data are shown for three contact cutoffs (see Methods). The graphs, arranged from top to bottom, display cluster sizes for 10, 20, and 50 CTD molecules. The three replicas are displayed using solid, dashed, and dotted lines, respectively. **(b)** Distributions of the radius of gyration for CTD^{cons} and the phospho-variants CTD^{pS5} and CTD^{pS7}, including their respective mean ± SD values. The distribution of CTD^{cons} is reprised as a dashed black line for ease of comparison. **(c)** Per-residue intramolecular interaction energy maps for the CTD constructs in **(b)**. The black arrows highlight repulsive energy contributions between the negatively charged phosphoresidues. SD is in range 0.002 to 4.998. Values matching or exceeding the limit of –0.3 kJ/mol are colored in dark red. Phosphorylated residues are coded by letter X. Results from all-atom simulations (panels **b** and **c**) were averaged over three replicas and di-heptad constructs.

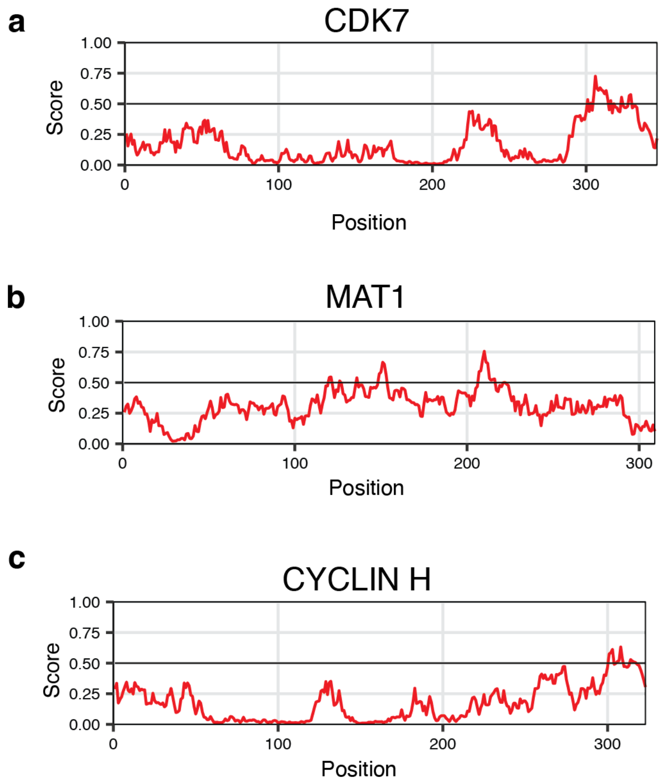

Supplementary Figure 14. IUPred2A[3,4] prediction of intrinsically disordered regions in the CDK7 complex for (a) CDK7, (b) MAT1, and (c) Cyclin H.

Supplementary Tables

Supplementary Table 1. Summary of mGFP-CTD constructs, protein constructs, sequences, and expression strains.

Construct Name	Heptad sequence	Expression strain	Expression vector	Solubility
mGFP-CTD	$(\mathrm{YSPTSPS})_{52}$*	*E. coli* BL21-AI(DE3) / *E. coli* JM103 (F-)	2Bc-T	ok
mGFP-CTD^{S2A}	$(\mathrm{Y\mathbf{A}PTSPS})_{52}$	*E. coli* BL21-AI(DE3)	2Bc-T	ok
mGFP-CTD^{S5A}	$(\mathrm{YSPT\mathbf{A}PS})_{52}$	*E. coli* BL21-AI(DE3)	2Bc-T	ok
mGFP-CTD^{S7A}	$(\mathrm{YSPTSP\mathbf{A}})_{52}$	*E. coli* BL21-AI(DE3)	2Bc-T	ok
mGFP-CTD^{T4G}	$(\mathrm{YSP\mathbf{G}SPS})_{52}$	*E. coli* BL21-AI(DE3)	2Bc-T	ok
mGFP-CTD^{S2,5A}	$(\mathrm{Y\mathbf{A}PT\mathbf{A}PS})_{52}$	*E. coli* BL21-AI(DE3)	2Bc-T	ok
mGFP-CTD^{S2,7A}	$(\mathrm{Y\mathbf{A}PTSP\mathbf{A}})_{52}$	*E. coli* BL21-AI(DE3)	2Bc-T	ok
mGFP-CTD^{S5,7A}	$(\mathrm{YSPT\mathbf{A}P\mathbf{A}})_{52}$	*E. coli* BL21-AI(DE3)	2Bc-T	ok
mGFP-CTD^{S2,5,7A}	$(\mathrm{Y\mathbf{A}PT\mathbf{A}P\mathbf{A}})_{52}$	*E. coli* BL21-AI(DE3)	2Bc-T	ok
mGFP-CTD^{Y1A}	$(\mathrm{\mathbf{A}SPTSPS})_{52}$	*E. coli* BL21-AI(DE3)	2Bc-T	ok
mGFP-CTD^{Y1F}	$(\mathrm{\mathbf{F}SPTSPS})_{52}$	*E. coli* BL21-AI(DE3)	2Bc-T	ok
mGFP-CTD^{P3G}	$(\mathrm{YS\mathbf{G}TSPS})_{52}$	*E. coli* BL21-AI(DE3)	2Bc-T	ok
mGFP-CTD^{P6G}	$(\mathrm{YSPTS\mathbf{G}S})_{52}$	*E. coli* BL21-AI(DE3)	2Bc-T	X**
mGFP-CTD^{P3,6G}	$(\mathrm{YS\mathbf{G}TS\mathbf{G}S})_{52}$	*E. coli* BL21-AI(DE3)	2Bc-T	X**
mCherry-CTD	$(\mathrm{YSPTSPS})_{52}$*	*E. coli* BL21-AI(DE3)	2Bc-T	ok
CTD (w/o tag)	$(\mathrm{YSPTSPS})_{52}$*	*E. coli* BL21-AI(DE3)	2S-T	ok
PIN1	-	*E. coli* BL21- CodonPlus (DE3) -RIPL	pMCSG7	ok
PIN1^{C113S}	-	*E. coli* BL21- CodonPlus (DE3) -RIPL	pMCSG7	ok
PPIA	-	*E. coli* BL21(DE3)	2BT	ok
PPIA^{R55A}	-	*E. coli* BL21(DE3)	2BT	ok
the CDK7 complex	-	*Trichoplusia ni* High Five (TM) insect cells	438B	ok

* CTD heptad contains variations in several repeats as in the wild-type CTD sequence.
** Expressed only in insoluble form under multiple conditions; purified under denaturing conditions and subsequent multiple refolding strategies failed.

Supplementary Table 2. List of oligonucleotides used in this study

Name	Sequence	
T7F	TAATACGACTCACTATAGGG	Sequencing
T7R	GCTAGTTATTGCTCAGCGG	Sequencing
mGFP_CTD_f	TTTAAGAAGGAGATATAGTTCATGAGCAAAGGCGAAGAACT	mGFP-CTD and the variants preparation forward
mGFP_CTD_r	GGATTGGAAGTAGAGGTTCTCATTT TCTTCATCACTATC	mGFP-CTD and the variants preparation reverse
mCherry_CTD_f	TTTAAGAAGGAGATATAGTTCATGATGGTGAGCAAAGGTGAAGAGGAT	mCherry-CTD preparation forward
mCherry_CTD_r	GGATTGGAAGTAGAGGTTCTCATTTTCTTCATCACTATCATC	mCherry-CTD preparation reverse
CTD (w/o tag)	TACTTCCAATCCAATGCATATAGCCCGACCAGTCCG	CTD (w/o tag) preparation forward
CTD (w/o tag)	TTATCCACTTCCAATGTTATTATCAATTTTCTTCATCACTATCATC	CTD (w/o tag) preparation reverse
PPIA_f	TACTTCCAATCCAATGCAATGGTCAACCCCACCGTG	PPIA preparation forward
PPIA_r	TTATCCACTTCCAATGTTATTATTATTCGAGTTGTCCACAGTC	PPIA preparation reverse
PPIA_R55A_f	CTGCTTTCACGC GATTATTCCAGGG	$PPIA^{R55A}$ preparation forward
PPIA_R55A_r	GAACCCTTATAACCAAATC	$PPIA^{R55A}$ preparation reverse
PIN1_C113S_f	GGCCTCACAGTTCAGCGATCCCAGCTCAGCCAAGGCCAGGGG	$PIN1^{C113S}$ preparation forward
PIN1_C113S_r	CCCCTGGCCTTGGCTGAGCTCCATCGCTGAACTGTGAGGCC	$PIN1^{C113S}$ preparation reverse
pRMS237	TACTTCCAATCCAATGCAATGGCTCTGGACGTGAAG	Amplification of CDK7 forward
pRMS238	TTATCCACTTCCAATGTTATTAAAAAATTAGTTTCTTGGGCAATCC	Amplification of CDK7 reverse
pRMS239	TACTTCCAATCCAATGCAATGGACGATCAGGGTTGC	Amplification of MAT1 forward
pRMS240	TTATCCACTTCCAATGTTATTAACTGGGCTGCCAGAAAAG	Amplification of MAT1 reverse
pRMS241	TACTTCCAATCCAATGCAATGTACCACAACAGTAGTCAG	Amplification of CCNH forward
pRMS242	TTATCCACTTCCAATGTTATTAGAGAGATTCTACCAGGTCG	Amplification of CCNH reverse

Supplementary Table 3. Summary of MD simulations. **P*** and **HYP*** represent prolines and hydroxyprolines in *cis* isomerization state, while **p** indicates phosphorylated amino acids. Information such as the number of simulated CTD constructs and replicas, resolution, and initial configurations are provided.

Construct Name	Heptad Sequence	Simulation Type
CTD^{cons}	YSPTSPS	**1, 2, 3**
$CTD^{\mathit{cis}P3}$	YS**P***TSPS	**1**
$CTD^{\mathit{cis}P6}$	YSPTS**P***S	**1**
$CTD^{\mathit{cis}P3,6}$	YS**P***TS**P***S	**1**
$CTD^{consHYP}$	YS**HYP**TS**HYP**S	**1**
$CTD^{\mathit{cis}HYP3,6}$	YS**HYP***TS**HYP***S	**1**
CTD^{Y1A}	**A**SPTSPS	**1, 2, 3, 4**
CTD^{Y1F}	**F**SPTSPS	**1, 2, 3**
CTD^{S2A}	Y**A**PTSPS	**1, 2, 3, 4**
CTD^{T4G}	YSP**G**SPS	**1, 2, 3, 4**
CTD^{S7A}	YSPTSP**A**	**1, 2, 3, 4**
CTD^{pS5}	YSPT**pS**PS	**1, 2, 4**
CTD^{pS7}	YSPTSP**pS**	**1, 2, 4**

1: Two copies of di-heptad CTD molecules at atomistic resolution. Run in triplicates.

2: Single chain CG simulation of CTD molecules composed of 52 heptad repeats.

3: Condensed phase CG simulation. 10, 20, 50, or 80 randomly distributed copies of CTD molecules composed of 52 heptad repeats. Run in triplicates.

4: Condensed phase CG simulation. Preformed droplets made of 10, 20, or 50 copies of CTD molecules composed of 52 heptad repeats. Run in triplicates.

Supplementary Table 4. Radii of gyration (Rg) and abundancy of protein clusters containing 70, 80, 90, and 100 percent of the total CTD molecules for CTD^{cons} and the CTD^{Y1F} variant in condensed phase CG simulations. From top to bottom, the data are shown for simulations with 10, 20, 50, and 80 CTD molecules. Abundancy and Rg values respectively represent the total sum and average (with SD) over three replicas. N.D. stands for “Not Defined". Periodic boundary conditions were fixed using FixBox algorithm[5].

	**Variant**	**70%**		**80%**		**90%**		**100%**	
		**Rg (nm)**	**Abundancy**	**Rg (nm)**	**Abundancy**	**Rg (nm)**	**Abundancy**	**Rg (nm)**	**Abundancy**
**10X**	**CTD^{cons}**	7.15 ± 0.13	6139	7.65 ± 0.4	6029	8.21 ± 1.39	8348	8.43 ± 1.22	1555
	**CTD^{Y1F}**	7.00 ± 0.36	7045	7.72 ± 0.68	2567	8.25 ± 0.48	618	9.70 ± 0.00	7
**20X**	**CTD^{cons}**	9.07 ± 0.12	2774	9.19 ± 0.09	5301	9.48 ± 0.33	5965	9.72 ± 0.23	760
	**CTD^{Y1F}**	8,97 ± 0.55	859	8.75 ± 0.18	3957	8.94 ± 0.16	6398	9.57 ± 0.87	3285
**50X**	**CTD^{cons}**	16.56 ± 1.21	114	17.14 ± 0.10	14	N.D.	N.D.	N.D.	N.D.
	**CTD^{Y1F}**	14.15 ± 0.54	2253	14.85 ± 0.88	2259	15.77 ± 1.76	667	N.D.	N.D.
**80X**	**CTD^{cons}**	N.D.	N.D.	N.D.	N.D.	N.D.	N.D.	N.D.	N.D.
	**CTD^{Y1F}**	19.19 ± 0.24	674	19.54 ± 0.09	165	19.67 ± 0.0	4	N.D.	N.D.

Supplementary Methods

Mass spectrometry

Sample preparation, proteolysis
To improve the sequence coverage, three different proteases were applied: trypsin, pepsin and alpha-lytic protease from lysobacter (WALP). Samples in a volume of 1 µl were mixed with 50 ng of trypsin/ WALP/pepsin and subjected to 2h/2h/1h proteolysis at 40°C/37°C/37°C in 50 mM ammonium bicarbonate/50 mM ammonium bicarbonate/10 mM HCl, respectively.

Sample preparation, MALDI-TOF MS
Samples in a volume of 0.6 µl were mixed with 2.4 µl of the MALDI matrix solution (12.5 mg/ml ferulic acid in acetonitrile:formic acid:water 50:33:17 v/v mixture for intact proteins, saturated alpa-cyano-4-hydroxycinnamic acid in acetonitrile:trifluoroacetic acid:water 50.0:2.5:47.5 v/v mixture for protein digests, or 10 mg/ml 5-methoxysalicylic acid in acetonitrile:water 1:1 v/v mixture for amino acids), 0.6 µl of the mixture was spotted onto a stainless steel sample plate, and allowed to dry at laboratory temperature.

Sample preparation, LC-MS/MS analysis of protein digests
The digests were vacuum-dried and re-constituted in 1% formic acid and 0.001% polyethylene glycol.

MALDI-TOF MS intact mass analysis of intact proteins
MALDI-TOF mass spectra measurements were carried out using an Ultraflextreme instrument (Bruker Daltonik, Bremen, Germany) operated in linear positive mode under FlexControl 3.4 software. External calibration of the mass spectra was performed using signals of lysozyme standard. The laser was set to 120 % of the threshold laser power for a particular type of sample. Within an individual sample spot, the laser was directed manually, where a minimum of 500 and a maximum of 2000 shots were obtained. Mass spectra were processed using Flex Analysis (version 3.4; Bruker Daltonik).

MALDI-TOF MS/MS intact mass analysis of protein digests
MALDI-TOF mass spectra measurements were carried out using an Ultraflextreme instrument (Bruker Daltonik, Bremen, Germany) under FlexControl 3.4 software. External calibration of the mass spectra was performed using a mixture of synthetic peptide standards covering the m/z range 1000-3500 Da. The laser was set to 120 % of the threshold laser power for a particular type of sample. Peptide maps were acquired in reflectron positive mode with 1000 laser shots. Peaks within 700 – 4000 Da mass range and minimum S/N 10 were picked out for MS/MS analysis employing LID-LIFT arrangement with 1000 laser shots for each peptide.

LC-MS/MS intact mass analysis of protein digests
LC-MS/MS analyses of peptide mixtures coming from digestions were done using RSLCnano system (Thermo Fisher Scientific) on-line connected to Impact II Ultra-High Resolution Qq-Time-Of-Flight mass spectrometer (Bruker, Bremen, Germany). The peptides were separated by using an Acclaim Pepmap100 C18 column (2µm particles, 75 µm × 500 mm; Thermo Fisher Scientific, Waltham, MA, USA) by the following gradient program (mobile phase A: 0.1% FA in water; mobile phase B: 0.1% FA in 80% acetonitrile; flow rate 300 nl/min): the gradient elution started at 2% of mobile phase B and increased from 2 % to 60 % during the first 25 min and then increased to 95 % of mobile phase B for the final 5 min. Equilibration of the analytical column was done before sample injection into sample loop. NanoBooster was filled with acetonitrile and nanoBooster pressure was set to 0.2 Bar. MS data were acquired in a data-dependent strategy with 3 s long cycle time. Mass range was set to 150-2200 m/z and

precursors were selected from 300-2000 m/z. Active exclusion was enabled for 2 min after one MS/MS spectra acquisition with Reconsider Precursor option enabled (intensity ratio of 3). Precursor charge state was monitored with exclusion of singly charged precursors. Acquisition speed of MS and MS/MS scans was 2 Hz and 4-16 Hz, respectively. Speed of MS/MS spectra acquisition was based on precursor intensity (low and high absolute thresholds were 2,500 and 25,000 cts. per 1,000 summations, respectively). Default CID collision energies and isolation widths with respect to precursor charge and m/z were used.

MALDI-TOF MS data processing
Mass spectra were processed (smoothing, baseline subtraction, peak picking) using Flex Analysis (version 3.4; Bruker Daltonik).

LC-MS/MS data processing
The processing of the LC-MS/MS data including recalibration, compounds detection, charge deconvolution and further MS/MS data analysis was carried out in DataAnalysis software (4.2 SR1; Bruker).

Supplementary References:

1. Pettersen, E. F. *et al.* UCSF ChimeraX: Structure visualization for researchers, educators, and developers. *Protein Sci* **30**, 70–82 (2021).
2. Schindelin, J. *et al.* Fiji: an open-source platform for biological-image analysis. *Nat Methods* **9**, 676–82 (2012).
3. Erdős, G. & Dosztányi, Z. Analyzing Protein Disorder with IUPred2A. *Curr Protoc Bioinformatics* **70**, e99 (2020).
4. Mészáros, B., Erdős, G. & Dosztányi, Z. IUPred2A: context-dependent prediction of protein disorder as a function of redox state and protein binding. *Nucleic Acids Res* **46**, W329–W337 (2018).
5. Baptista, A. M., da Rocha, L. & Campos, S. R. R. FixBox: A General Algorithm to Fix Molecular Systems in Periodic Boxes. *J Chem Inf Model* **62**, 4435–4447 (2022).
